# Complementary and Alternative Medicine for Dysmenorrhea Caused by Endometriosis: A Review of Utilization and Mechanism

**DOI:** 10.1155/2021/6663602

**Published:** 2021-07-02

**Authors:** Ying Guo, Fang-Yuan Liu, Ying Shen, Jia-Yue Xu, Liang-Zhen Xie, Shi-Ying Li, Dan-Ni Ding, Dan-Qi Zhang, Feng-Juan Han

**Affiliations:** ^1^Department of Obstetrics and Gynecology, The First Affiliated Hospital of Heilongjiang University of Chinese Medicine, Harbin 150040, China; ^2^Department of Obstetrics and Gynecology, Heilongjiang University of Chinese Medicine, Harbin 150040, China

## Abstract

Endometriosis (EM) is a common and benign estrogen-dependent gynecological disorder among women of reproductive age, and secondary dysmenorrhea is one of the more severe symptoms. However, the mechanism behind the development of dysmenorrhea is poorly understood, and there is a lack of effective methods for diagnosing and treating EM dysmenorrhea. In this regard, complementary and alternative medicine (CAM) has recently come into widespread use due to its limited adverse reactions and high efficiency. This review updates the progress of CAM in the treatment of EM dysmenorrhea and seeks to identify the therapeutic efficacy as well as the mechanisms behind these effects based on the available clinical and experimental studies. According to the literature, CAM therapy for EM dysmenorrhea, including herbs (herbal prescriptions, extracts, and patents), acupuncture, and Chinese herbal medicine enema (CHM enema), is effective for relieving dysmenorrhea with fewer unpleasant side effects when compared to hormonal and surgical treatments. In addition, we discuss and analyze the existing gaps in the literature. We hope to provide some instructive suggestions for clinical treatment and experimental research in the future.

## 1. Introduction

**Figure 1:**
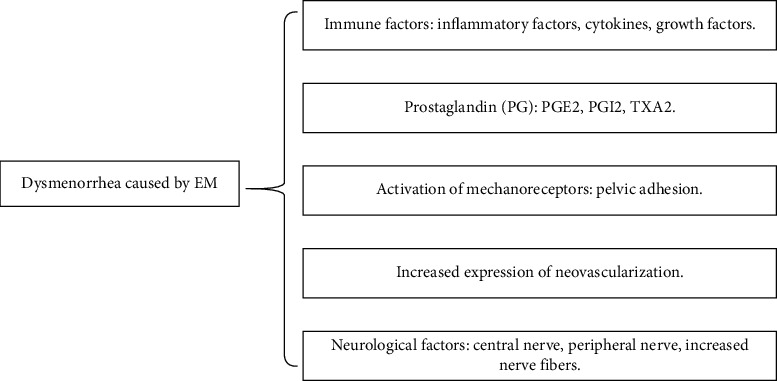
Risk factors for EM dysmenorrhea.

Endometriosis (EM) is defined as endometrial tissue, including glands and stroma, which grows abnormally in locations outside the uterus. EM is the second most common benign female genital disease after uterine myoma [1]. About 15% of women of reproductive age suffer from EM [2], but the actual incidence is higher because this figure is only for symptomatic cases [3]. EM is characterized by chronic pelvic pain, secondary dysmenorrhea, dyspareunia, infertility, abnormal uterine bleeding, and so forth. As one of the more severe symptoms, secondary dysmenorrhea refers to pain or cramps before or during menstruation. In 78.7% of women with EM, dysmenorrhea is the symptom that led to their diagnosis and severely reduces their quality of life [4]. The pathophysiology of EM dysmenorrhea may include immune factors [5], excessive production of prostaglandin causing ischemia [6], activation of mechanoreceptors [7], increased neovascularization [8], neurological factors [9, 10], and so forth (Figure 1).

Treatment for EM dysmenorrhea can be medical and/or surgical. For mild cases, nonsteroidal anti-inflammatory drugs play an important role in alleviating menstrual pain. However, for cases who are unresponsive to these drugs, they can be treated by hormone replacement therapy, including progestogenics, gestrinone, danazol (androgen derivatives), and gonadotropin-releasing hormone agonists to alleviate pain [11]. Surgical methods are also needed for those patients who cannot accept oral medicines. About 18% of women with dysmenorrhea are unresponsive to nonsteroidal anti-inflammatory drugs [12], and women receiving hormone replacement therapy are at increased risk for some cancers and cardiovascular diseases. Moreover, EM has a high recurrence rate. Thus, there is an urgent need to develop better strategies for treating EM.

Complementary and alternative medicine (CAM) refers to various health care and treatment systems that are independent of Western medicine, such as traditional Chinese medicine (TCM), qi gong, and homeopathy [13]. The acknowledged advantages of CAM are that they are natural, convenient, and affordable, and thus, CAM has gained favor with many nationalities and peoples [14]. CAM is an effective strategy to alleviate the pain associated with many diseases, and both academic researchers and governmental agencies have started to incorporate CAM recommendations into chronic pain management strategies [15]. Therefore, this review updates the progress of CAM in the treatment of EM dysmenorrhea. According to the available literature, CAM methods for EM dysmenorrhea mainly include herbal products, acupuncture and moxibustion, and CHM enema (Table 1).

## 2. Search Strategy and Selection Criteria

The literature search was performed using the key words “Endometriosis,” “Dysmenorrhea,” “Menstrual Pain,” “Traditional Chinese Medicine,” “Complementary and alternative medicine,” and “Herbal Extracts” in PubMed, CNKI, VIP, Web of Science, Embase, and Wanfang from the date of establishment of the database to 2021. References in the identified studies were also searched to identify relevant literature. A total of 1,060 articles were identified in the search, and 116 articles were deemed potentially relevant. Among them, there are 51 articles on Chinese products, 30 articles on acupuncture and moxibustion, and 15 articles on CHM enema.

## 3. Herbal Products

Herbal products mainly include compound preparations (consisting of two or more herbs) in which all of the medicines are boiled together in water and then processed into herbal extracts, pills, or capsules (patents). Tables 2 and 3 list the most commonly used herbs for treating EM dysmenorrhea. In China, compound preparations are generally more effective than other treatment methods. Researchers have suggested that the potential mechanism of herbal products on EM dysmenorrhea is through (1) reducing the viscosity of whole blood, improving pelvic microcirculation, and adjusting the expression level of related factors [16, 17], (2) inhibiting the expression of inflammatory factors such as interleukin-1 (IL-1) and interleukin-6 (IL-6) [18], (3) reducing the expression of prostaglandin E2 (PGE2), prostaglandin f 2*α* (PGF2*α*), and nerve growth factor (NGF) in patients with EM dysmenorrhea [19], and (4) inhibiting uterine smooth muscle activity and relieving uterine smooth muscle spasm [20].

### 3.1. Herbal Decoction Therapies

There are several common decoctions used to treat EM dysmenorrhea in China, including Shaofu Zhuyu decoction (SZD), Wengjing decoction (WJD), Xuefu Zhuyu decoction (XZD), and Danggui Sini decoction (DSD). Many clinical and experimental studies have confirmed that Chinese herbal decoctions play an important role in the treatment of EM dysmenorrhea.

#### 3.1.1. SZD

SZD comes from QR Wang's *Correction of Errors in Medical Works*, which was written in the Qing dynasty and is still widely used in the clinic. In the theory of TCM, SZD is mainly used in EM dysmenorrhea due to cold blood stasis. SZD can reduce the expression of tumor necrosis factor (TNF-*α*), IL-6, and interleukin-8 (IL-8); the mRNA expression of extracellular regulated protein kinases (ERK), vascular endothelial growth factor (VEGF), and matrix metalloprotein-9 (MMP-9); and the protein expression of nuclear factor-*κ*B (NF-*κ*B), mitogen-activated protein kinase (MAPK), and MAPK-ERK kinase (MEK), thus influencing the MAPK/ERK signaling pathway as a way to treat EM [21]. SZD can inhibit proliferation in the eutopic endometrium by inducing apoptosis in eutopic endometrial stromal cells in a rat model of EM [22]. A randomized controlled trial (RCT) showed that SZD could relieve dysmenorrhea more effectively than ibuprofen capsules. The total effective rates were 90% and 70%, respectively, after 3 months of treatment in a study group of 20 patients [23]. SZD can reduce cell proliferation, increase apoptosis, and inhibit angiogenesis and hypoxia inducible factor-1*α* (HIF-1*α*) expression, and SZD seems to be helpful in preventing the recurrence of EM after surgery [24].

#### 3.1.2. WJD

WJD has been widely used to treat various disorders in China since the Han dynasty. WJD has an inhibitory effect on the growth of ectopic endometrium in a mouse model of EM and reduces the levels of inflammatory factors and improves the inflammatory response [25]. In the clinic, the results have been similar to those in animal trials. Compared with mifepristone, WJD could significantly lower serum CD4^+^, CD4^+^/CD8^+^, IL-4, and IL-10 levels to relieve pain [26]. In addition, Tang and Wu [27] selected 60 patients who were randomly divided equally into the treatment group (WJD) and control group (ibuprofen sustained-release capsules) for 3 months. The total effective rate of the treatment group was 90.0%, which was significantly higher than 76.7% in the control group, and the visual analogue scale (VAS) scores of the two groups were significantly reduced. WJD appears to be effective in treating EM dysmenorrhea, and thus should be considered for clinical applications.

#### 3.1.3. XZD

XZD is also from Wang's *Correction of Errors in Medical Works*. In TCM, most doctors agree that EM dysmenorrhea's pathogenesis is blood stasis, so XZD or SZD is widely applied [28]. XZD acts on multiple EM targets and participates in regulating multiple signaling pathways through its influence on TNF and estrogen [29]. Fu and Xie [30] treated 94 patients with EM dysmenorrhea with XZD or mifepristone, and the total effective rates were 89.4% and 78.7%, respectively. XZD can also reduce the VAS score of patients with EM and can relieve pain [31].

#### 3.1.4. DSD

DSD contains angelica, cinnamon, and asuram and is a common decoction for treating EM dysmenorrhea. According to modern pharmacological research [32], angelica promotes the proliferation of hematopoietic progenitor cells related to antithrombotic and antiplatelet aggregation. Cinnamon contains coumarins, organic acids, volatile components, and other chemical components that exhibit antiviral, antiallergic, anti-infective, diuretic, antipyretic, and analgesic properties. Asarum has anti-infection, vasodilator, heart strengthening, antipyretic, and analgesic effects. Liu [33] applied DSD combined with different doses of mifepristone to evaluate the postoperative curative effect on EM and found that 5 mg of mifepristone combined with DSD could reduce serum levels of CA-125 and sex hormones and ked to a lower recurrence rate compared to 10 mg mifepristone combined with DSD. Li et al. [34] treated EM dysmenorrhea patients with DSD and reported an overall efficiency of 89.19%.

#### 3.1.5. Other Herbal Decoction Therapies

Some reports of treating EM with Danggui Shaoyao Powder (DSP) showed that it could reduce serum progesterone levels, effectively regulate serum PGE2 and PGF2*α* concentrations, and significantly alleviate pain in EM dysmenorrhea patients [35, 36]. In addition, Bushen Zhuyu decoction (BSZYD) is 94.12% effective in treating EM, and IL-6, IL-8, and TNF-*α* levels are significantly reduced after treatment, indicating that BSZYD can improve EM dysmenorrhea and the pelvic microcirculation of patients with dysmenorrhea, regulate the expression level of related factors, and relieve pain [37]. Chai and Wang [38] treated EM dysmenorrhea patients with Gexia Zhuyu decoction (GZD), and it was proposed that GZD can reduce serum CA-125 levels, plasma viscosity, and the erythrocyte sedimentation rate. A rat trial showed that Qingre Huayu decoction could reduce the mRNA expression of NF-*κ*B, VEGF, and cyclooxygenase-2 (COX-2), thus inhibiting inflammation and angiogenesis [39]. Various herbal mixtures are used, but only the most commonly used are discussed in this section.

### 3.2. Herbal Extract Therapies

Several herbal extracts are commonly used to treat EM dysmenorrhea in China, including rhizoma zedoariae water decoction, triterpenoid saponin, and emodin. All of these may affect cell proliferation, apoptosis, angiogenesis, immunity, and the inflammatory microenvironment through multiple pathways, thus playing an important role in EM dysmenorrhea.

#### 3.2.1. Rhizoma Zedoariae Water Decoction

Rhizoma zedoariae water decoction is from the dried tuber of *Curcuma phaeocaulis valeton* (the traditional method is to add water and then boil to concentrate to 2 g/mL), and this can inhibit the growth of ectopic endometrium in a rat model of EM [40]. The mechanism is related to the downregulation of JAK2, STAT3 phosphorylation, and protein overexpression and to the reduction of JAK2 and STAT3 levels in ectopic endometrial tissues. STAT3 signaling has been shown to stimulate cell proliferation, inhibit apoptosis, promote angiogenesis, mediate immune evasion, and participate in tumorigenesis and development, and the interaction between JAK and STAT3 promotes these cellular responses [41]. Similarly, studies in rats also demonstrated that curcumenol, the main active ingredient in zedoary, has antibacterial, anti-inflammatory, antioxidation, antitumor, antiplatelet aggregation, and antithrombotic activities and showed that curcumenol significantly reduces the levels of human macrophage chemoattractant protein-1 (MCP-1), migration inhibition factor (MIF), TNF-*α*, IL-1*β*, and IL-6 in the peritoneal fluid in the rat model of EM and has obvious inhibitory effects on inflammatory reactions in the abdominal microenvironment [42].

#### 3.2.2. Triterpenoid Saponin

Triterpenoid saponin types are more widely distributed in nature than steroidal saponins and are predominantly found in Leguminosae, Araliaceae, Polygalaceae, and Cucurbitaceae such as ginseng, cohosh, Panax, and licorice. According to some rat trials [43, 44], the mechanism of action of triterpenoid saponins may be related to the inhibition of NF-*κ*B activity, leading to increased IFN-*γ* cytokine secretion and decreased expression of cytokines such as IL-6 and TNF-*α*, thus inhibiting the formation and development of lesions. These cytokines, which are immune factors, play important roles in EM occurrence and development.

#### 3.2.3. Emodin

As the main component of many Chinese herbal medicines, emodin is a cymbidium compound with many biological activities such as immunosuppressive, antibacterial, anti-inflammatory, and antitumor properties [45]. By regulating the integrin-linked kinase (ILK) pathway, which in turn regulates cell survival, proliferation, and invasion by regulating various signal transduction pathways, emodin induces the development of endometrial stromal cells' MET, thereby inhibiting their migration and invasion [46, 47].

#### 3.2.4. Other Herbal Extracts

Other herbal extracts also play particular roles in treating EM, and the mechanism of five Chinese medicine monomers on uterine smooth muscle was reported, including *Atractylodes macrocephala*, rhein, alizarin, turmeric, and corydalis [48]. Their functions are mainly through H1 receptors and L-type calcium channels, and the H1 receptor functions in prostaglandin (PG) formation and release. To sum up, herbal extracts play a vital role in treating EM dysmenorrhea and should be investigated further.

### 3.3. Herbal Patent Medicine Therapies

Herbal patent medicines are based on Chinese medicine theory using CHM as raw materials. These are processed into various dosage forms and provide new Chinese medicine forms for patients who find Chinese medicine decoctions to be inconvenient to take. Chinese patent medicines are easy to carry and taste good, including Guizhi Fuling capsules (GFC), ELeng capsules (ELC), Dan Bie capsules (DBC), Sanjie Analgesic capsules (SAC), and Dane Fukang paste (DFP).

#### 3.3.1. GFC

GFC has been shown to be effective in treating EM dysmenorrhea. The results of modern pharmacological studies show that the active ingredients in GFC have anti-inflammatory, analgesic, and immune-regulating effects [49–51]. Clinical studies have shown that GFC can effectively reduce the levels of serum CA-125 and CA-199 in patients with EM dysmenorrhea and at the same time can effectively improve the TCM syndromes of patients, including menstrual cycle and menstrual volume [52]. A clinical study conducted by Tao and Yu showed that GFC could lower the levels of MEK-2, ERK-5, p-ERK, VEGF, T-cad, and VE-cad, demonstrating that GFC can significantly inhibit the cell proliferation and differentiation of EM by inhibiting the activity of MEK and ERK proteins and blocking the signal transduction pathways between cells, thereby inhibiting the abnormal proliferation of endometrial cells [53].

#### 3.3.2. ELC

ELC can significantly inhibit EM tissue development in rats. Its mechanism of action may be related to inhibition of VEGF and its receptor, which are proangiogenic factors, with the strongest action and highest specificity for inhibiting angiogenesis in ectopic lesions [54]. A retrospective study found that ELC application before surgery can improve the pelvic microenvironment and make it more conducive to surgery. The mechanism behind this is likely through inhibition of the expression of soluble intercellular molecule-1 (sICAM-1), which is a member of the immunoglobulin superfamily of adhesion molecules and functions in damage repair, inflammation, immune response, and tumor metastasis. In addition, ELC can block the adhesion of ectopic lesions. Simultaneously, reduction of sICAM-1 reduces the promotion of vascular proliferation and adhesion formation [55]. Interestingly, Xu et al. [56] showed that the level of sICAM-1 in patients treated with ELC and mifepristone was higher than that in the control group treated with mifepristone alone, and the specific mechanism behind this effect needs further study. Huang et al. [57] performed an RCT using ELC with EM patients, and the total effective rate of dysmenorrhea was decreased from 84% to 48%.

#### 3.3.3. DBC

DBC can reduce the concentration of PGF2*α* and PGE2 in EM patient's serum, which can relax the small blood vessels, reduce pelvic stasis, improve the pelvic microcirculation, and relieve pain according to a rat model [58, 59]. DBC has many functions such as anti-inflammatory and analgesic effects and improving blood rheology and microcirculation to treat EM dysmenorrhea [60].

#### 3.3.4. SAC

The active ingredients in SAC have significant biological activities such as anti-inflammatory, analgesic, and hormone-like effects. They can act on inflammation, cell invasion, metastasis, coagulation, and smooth muscle contraction, thereby improving endometrial blood vessel function, improving the blood perfusion of the pelvis and uterus, inhibiting smooth muscle contraction, and regulating the immune inflammatory response [61, 62]. SAC has been shown to treat EM more effectively than danazol with a total effective rate of 92.9% vs. 77.5% [63].

#### 3.3.5. DFP

Clinical studies have shown that DFP promotes blood circulation, removes blood stasis, soothes the liver, regulates qi, regulates menstruation, and relieves pain [64]. Ye and Wu [65] divided 150 patients with EM into an observation group and a control group with 75 cases each according to a random number table. The control group was treated with gestrinone, and the patients in the observation group were treated with DFP. The total effective rate in the observation group was 95.45%, which was significantly higher than the effective rate of 81.82% in the control group. Studies have shown that DFP may treat EM dysmenorrhea by regulating the body's immunity and inhibiting the proliferation, migration, and invasion of endometrial cells [66].

## 4. Acupuncture and Moxibustion Treatment

Acupuncture has been practiced for more than 3000 years in China, and it spread throughout Europe and America from the sixteenth to the nineteenth centuries. The history of acupuncture research was initiated in the eighteenth century and has developed rapidly since then [67]. Acupuncture has attracted increasing attention as a safe and easy-to-perform treatment [68]. Acupuncture has evolved from its original methods to include moxibustion, acupoint catgut implantation therapy, electroacupuncture, auricular acupoint treatment, and acupuncture combined with other therapies [69]. All of these can effectively relieve the symptoms of dysmenorrhea caused by EM (Tables 4 and 5). Based on a large number of clinical and animal experiments, the different mechanisms of acupuncture treatment for dysmenorrhea include relaxation of the meridians and promotion of blood circulation, modulation of immunity, activation of various neurotransmitters, reduction of VEGF, and regulation of abnormal prostaglandins, *β*-endorphin, dynorphin, electrolytes, and substance P levels in the body [70, 71]. Therefore, it plays a significant role in treating diseases.

### 4.1. Acupuncture Treatment

Acupuncture can relieve pain in the central and peripheral regions by activating various neurotransmitters or modulators, including serotonin, norepinephrine, and adenosine [72]. Xu et al. [73] carried out a systematic review and meta-analysis to determine the effects of acupuncture on treating EM-related pain. Patients in the intervention group were treated with acupuncture, and patients in the control group were treated with sham acupuncture, TCM, or western medicine. The results showed that the total effective rate of the intervention group reached 95%, and acupuncture had obvious advantages in relieving pain, reducing CA-125 concentration, and improving clinical symptoms. Shen and Lu [74] treated 50 EM patients with acupuncture or mifepristone and showed that acupuncture significantly reduced the extent of dysmenorrhea and reduced serum CA-125 levels. Xiao et al. [75] analyzed the relevant literature on modern acupuncture and moxibustion treatments for EM (Table 6).

### 4.2. Moxibustion Treatment

Moxibustion is the traditional method of burning the dried leaves of the mugwort plant (*Artemisia vulgaris*) to stimulate acupuncture points [76]. Correspondingly, moxibustion's effects are associated with properties from burning the dried herb, including the thermal stimulation. Chen et al. [77] sought evidence to confirm moxibustion's effect. Fifty-four EM patients were randomly divided into the moxibustion treatment group and the ibuprofen sustained-release capsule control group. The VAS scores and the days of dysmenorrhea were decreased in the treatment group and were less than those in the control group (*P* < 0.05).

### 4.3. Acupuncture Combined with Moxibustion Treatment

Acupuncture combined with moxibustion is a common practice in TCM and can effectively stimulate the regulatory function of meridians and collaterals, thus improving local blood stasis [78]. Mu [79] treated 42 patients with acupuncture combined with moxibustion. The acupuncture treatment was performed in the Zhongwan (CV12), Xiaguan (ST7), Qihai (RN6), Zhongji (CV3), Guanyuan (CV4), Qixue (KI13), Shuidao (S28), Taixi (K13), and Zigong (EX-CA1) acupoints. The acupuncture needles were retained for 30 min after de qi, and moxibustion was performed with ai zhu after acupuncture, which was effective in 29 patients.

Warming needle moxibustion, which combines acupuncture and moxibustion, involves wrapping moxa on the needle handle (or fixing a suitable length of moxa stick onto the needle handle) and igniting it during the needle retention process [80]. The needle body transfers the heat into the acupoint to treat disease by warming the meridians and promoting qi and blood circulation. It has a wide range of indications and is often used in the treatment of pain. Pan [81] treated 35 EM patients with existing dysmenorrhea with warming needle moxibustion at the following acupoints: Zusanli (ST36), Siman (KI14), Sanyinjiao (SP5), Qihai (RN6), Shuidao (RN9), Tianshu (ST25), and Zhongwan (RN12). After 3 months of treatment, the pain score had decreased significantly compared to the Western medicine group.

### 4.4. Acupuncture Combined with TCM

The combination of acupuncture with Chinese medicine is an important means of treating diseases. Acupuncture combined with Chinese medicine can reduce VEGF, a highly specific provascular endothelial cell growth factor, and this in turn promotes increased vascular permeability, extracellular matrix degeneration, and the migration, proliferation, and angiogenesis of vascular endothelial cells in a rat model of EM [82]. Another experiment in a rat model of EM showed that the combination of acupuncture and Chinese medicine could increase the levels of 6-keto-PGF1*α* and decrease the levels of thromboxane B2 (TXB2), thus regulating the imbalance of the two, which may be the mechanism through which acupuncture combined with Chinese medicine relieves the pain caused by EM [83]. Cui and Yang [84] performed a clinical study in which acupuncture was given one week prior to menstruation (acupoints: Guanyuan (CV4), Qihai (RN6), Zhongji (CV3), Zigong (EX-CA1), Sanyinjiao (SP5), Diji (SP8), and NeiYiJian)) and found that the clinical efficacy of acupuncture combined with Chinese medicine (86.67%) was higher than that of mifepristone (60.00%), and the uterine artery hemodynamics and EM-related serological parameters were reduced.

### 4.5. Acupoint Catgut Implantation Therapy

Acupoint thread-embedding therapy is based on acupuncture and moxibustion therapy. Absorbable protein filaments are implanted into acupoints to maintain the continuous stimulation of acupoints for a long time, which allows for the easy dissipation of local congestion [85]. Cong et al. [86] applied the catgut-embedding method at the Xuehai (SP10), Sanyinjiao (SP5), Diji (SP8), Zigong (EX-CA1), and Guanyuan (CV4) acupoints once every 2 weeks six consecutive times, resulting in tonifying the kidneys, warming the meridians, removing blood stasis, and clearing collaterals, with a total effective rate of 96.97%.

### 4.6. Electroacupuncture Treatment

Electroacupuncture is a modified form of acupuncture that uses electrical stimulation and is a widely used TCM therapy [87]. Direct stimulation can be carried out on the transtendon point through electric conduction, and the current can reach areas that acupuncture needles cannot reach, which can effectively improve blood circulation. Electroacupuncture activates the nervous system differently in healthy patients compared to those in pain, and it alleviates both sensory and effective inflammatory pain and inhibits inflammatory and neuropathic pain more effectively at 2–10 Hz than at 100 Hz. Electroacupuncture blocks pain by activating various bioactive chemicals through peripheral, spinal, and supraspinal mechanisms [88, 89]. Zhang and Li [90] treated patients with electroacupuncture (acupoints: Qihai (RN6), Guanyuan (CV4), Zhongji (CV3), Zigong (EX-CA1), Diji (SP8), Sanyinjiao (SP5), Hegu (LI4), Taichong (LR3)). After de qi, a G6805-I electronic pulse generator was attached to needles at the bilateral Zigong (EX-CA 1), Guanyuan (CV 4), and Zhongji (CV 3) acupoints, and a continuous wave was generated at a frequency of 70 Hz and an intensity of 3 mA. The control group was treated with mifepristone. The curative group's total effective rate was 94.4%, and the recurrence rate within a 1 year was low.

### 4.7. Acupuncture Combined with Acupoint Sticking Treatment

Acupoint sticking is the combination of acupoints and drugs, which can dredge channels and collaterals and promote blood circulation by stimulating the effects of drugs on the body's corresponding acupoints in order to achieve the treatment goal [91]. Chen et al. [92] treated 73 EM dysmenorrhea patients who were divided into the observation group (36 cases) with acupuncture combined with acupoint sticking (acupoints: Zhongji (CV3), Guanyuan (CV4), Zigong (EX-CA1), etc.) and the control group (37 cases) treated with Jiawei Mojie tablet. The long-term total effective rates for the treatment group and the control group were 97.1% and 69.4%, respectively, and the treatment group had good long-term effects and stable conditions.

### 4.8. Auricular Acupoint Treatment

Auricular acupuncture involves the stimulation of auricular points on the ear. According to TCM theory, there is a natural mode of essence in the human body [93], and a study showed that auricular points are effective in controlling pain and regulating immunity through multiple mechanisms [94]. Auricular acupuncture is easy and convenient, has good analgesic effects, and is long lasting [95]. Xiang et al. [96] found that the total effective rate of ear acupuncture in EM patients with mild-to-moderate dysmenorrhea was 91.9%.

Although doctors' understandings of dysmenorrhea caused by EM are different based on years of clinical practice, starting from basic theories of TCM four diagnostic parameters, syndrome differentiation, and individualized treatment plans can be made, which rely on the patient's, physical condition, ages, and flexible use of TCM. Acupuncture, patching, moxibustion, acupoint embedding, etc. are used as pain relief methods and have achieved good clinical results. TCM has unique advantages in treating EM; therefore, we should pay more attention to performing more research in order to provide more evidence-based treatments for this disease.

## 5. CHM Enema and Related Therapies

As an important part of the external treatment in TCM, CHM enema has been widely used in the clinic. CHM enema delivers TCM decoctions into the rectum from the anus so that the medicine can be retained in the intestine and the intestinal mucosa can absorb the drug to achieve the purpose of treating and preventing the disease [97]. The advantage of this therapy is that it can effectively avoid the “first elimination” effect of the liver and avoid the digestive effect of the gastrointestinal tract that can destroy the drug before it reaches the pelvic cavity and can alleviate the pain caused by EM [98]. CHM enema is often combined with oral Chinese medicine, microwave physiotherapy, and patch therapy in the treatment of EM dysmenorrhea, as listed in Table 7.

### 5.1. CHM Enema

The mechanism of CHM enema to treat EM dysmenorrhea may be by inhibiting the activation of NF-*κ*B in EMT cells, reducing the expression and secretion of regulated on activation in normal T-cell expressed and secreted (RANTES), thereby reducing inflammation and pain [99]. Yu and Li [100] treated patients suffering from EM dysmenorrhea with CHM enema (drugs: danshen 15 g, chishao 15 g, mudanpi 15 g, wulingzhi 15 g, yanhusuo 15 g, zaojiaoci 15 g, danggui 15 g, ezhu 15 g, muxiang 10 g, rougui 10 g, chenpi 10 g, and quanxie 3 g) or oral Sanjie analgesic capsules. After treatment, the serum CA-125 and VEGF levels decreased significantly compared with the control group, indicating a clear clinical effect of enema treatment on EM dysmenorrhea. Tian [101] treated 94 patients with EM-related pain who were randomized into two treatment groups with CHM enema (drugs: danshen 20 g, sanleng 15 g, ezhu 15 g, zaojiaoci 10 g, yimucao 15 g, and yanhusuo 20 g) or mifepristone. The efficiency rates were 89.4% and 78.7% for the treatment and control groups, respectively. In general, it appears that CHM enema can achieve twice the result with half the effort.

### 5.2. CHM Enema Combined with Oral CHM

In most Chinese hospitals, CHM enemas combined with oral CHM are the most commonly utilized treatments for dysmenorrhea caused by EM. Wu and Li [102] selected 86 patients with EM and divided them into control and observation groups using a random number table. The control group was given oral gestrinone capsules, and the observation group was given Jiawei Xuefu Zhuyu decoction both orally and as an enema. After 6 months of treatment, the observation group's total clinical effective rate was 95.35%, while that of the control group was 86.05%. The total clinical effective rate the observation group was significantly higher than that of control group. The difference in estradiol (E2), prolactin (PRL), and progesterone (P) hormone levels was statistically significant (*P* < 0.05). Lou [103] selected 92 patients with EM and randomly divided them into observation and control groups. The observation group was given oral CHM combined with CHM enema, while the control group was given mifepristone. Comparing the two groups of patients after treatment, the total effective rate in the Chinese medicine combined enema group was 91.30%, which was significantly greater than the effective rate of 73.91% in the control group.

### 5.3. CHM Enema Combined with Microwave Physiotherapy

Microwave physiotherapy has the advantages of simple, safe operation, and low side effects in treating gynecological diseases [104]. Microwaves are a kind of high-frequency electromagnetic wave with strong penetrability. The principle in using microwave's thermal effects is to expand local blood vessels, promote blood circulation, and improve local nutrition [105]. Microwave therapy uses the magnetocaloric effect of microwaves on the tissues to stimulate different areas around the rectal wall. The microwave treatment shrinks and softens the endometrial sac, accelerates blood circulation around the lesion, improves capillary permeability, and relieves smooth muscle spasms and thereby improves the patient's clinical symptoms [106]. Tang et al. [107] randomly divided 72 patients with EM into the observation and control groups. The observation group received microwave therapy with TCM ointments, while the control group was given danazol orally for 3 months. The total effective rate of TCM ointments combined with microwave treatment on dysmenorrhea was 94.40% compared to 77.80% for the controls.

### 5.4. CHM Enema Combined with Patch Therapy

The mechanism of patch therapy is induction and conduction through the meridian, and the drug is absorbed through the skin. This method allows the drug to remain on the local skin surface for a long time, stimulating the skin receptors and reaching the disease through the skin so that the drug is supplied continuously [108]. Chinese herbal enema combined with patch therapy can alleviate the clinical symptoms and reduce the level of IL-8 in EM patients. The mechanism of action in relieving pelvic inflammation may be by improving the local microenvironment of the uterine cavity and increasing pelvic blood circulation [109].

Wan et al. [110] randomly divided 64 patients with secondary dysmenorrhea into an observation group receiving CHM enema combined with patch therapy and a control receiving oral ibuprofen sustained-release capsules. The relief rate of dysmenorrhea in the treated group was significantly higher than that of ibuprofen control group (*P* < 0.05). Deng [111] used CHM retention enema and external application of Chinese medicine compared to CHM retention enema alone. The results showed that the total effective rate of CHM retention enema combined with external application of Chinese medicine was 93.48%, while for the control group it was 63.04%. Therefore, CHM enema combined with external application has good therapeutic effects for treating EM and has high safety for reducing the focus of disease, alleviating dysmenorrhea, and regulating the ovarian axis.

## 6. Other CAM Therapies

There are some other CAM therapies for dysmenorrhea caused by EM, including Chinese herbal soaking method, copper Bian stone scraping therapy, and foot massage [112]. The TCM dip stain method is to wet the affected area with gauze full of medicinal juice so that the medicinal juice can pass through the Xuanfu and enter the hair follicle, thereby promoting blood circulation, dredging collaterals, and relieving pain. Jiang et al. [113] selected 80 patients with EM and randomly divided them into observation and control groups. The observation group was treated with the TCM dip stain method, and the control group was treated with Shaofu Zhuyu granules. The dysmenorrhea score and total effective treatment rate in the observation group were significantly higher than those in the control group (*P* < 0.05). Traditional Gua Sha therapy has the functions of dredging meridians, promoting qi, and increasing blood circulation. A clinical study reported that using Zusanyin meridian curettage therapy to treat dysmenorrhea is effective [114], and copper Bian stone scraping therapy compared with traditional Gua Sha therapy has greater ability to regulate qi and blood circulation and has stronger local penetration [115]. Cong et al. [116] randomly divided 56 patients with EM dysmenorrhea into observation and control groups. The control group was treated with Guixiang Wenjing Zhitong capsules orally, and the observation group was treated with Cuban scraping therapy. After three courses of treatment, the observation group's total effective rate was 96.43%, which was significantly higher than the control group's 78.57%. There is still a lack of studies into the detailed mechanisms of foot massage. We will continue to focus on future avenues of research in this field.

## 7. Conclusions

There are increasing CAM therapies for secondary dysmenorrhea caused by EM, including herbs, acupuncture, and CHM enema. CAM therapies have been widely utilized because their curative effect is well accepted. These therapies can relieve pain, reduce the recurrence rate, and improve quality of life; however, they cannot fully eradicate the endometriotic lesions. In summary, the active principle of CAM therapies has a strong scientific foundation, and researchers have shown increased interest in this area of medical treatment. Standardizations of effective CAM therapies are still needed in order to increase the benefits of these alternative medical interventions for patients with EM dysmenorrhea throughout the world. In the future, larger samples and RCTs are needed to confirm the efficacy and safety of CAM for the treatment of EM dysmenorrhea and to provide new approaches for the management of EM dysmenorrhea.

## Figures and Tables

**Table 1:** General view of all therapeutic approaches.

Therapeutic approaches	Specifications	Efficacy	Precautions	Refs.
Herbal products	Appropriate TCM prescriptions are proposed according to TCM doctors' judgment.	Alleviating dysmenorrhea.	Allergy to drugs or contraindications.	[16–66]
Acupuncture and moxibustion	Use the appropriate acupoints or moxa-moxibustion therapy in light of the disease status of the patient. Most acupuncture treatments are 30 min (needling and auricular point), while moxibustion treatments last 40–50 min.	Alleviating dysmenorrhea.	Be careful of fainting conditions.	[67–96]
CHM enema	Ask patients to take the left lateral decubitus position. Put the boiled TCM herbal liquid into a 20 mL syringe, and wait for the temperature to reach 38–40°C. With a disposable catheter connection, slowly push the TCM herbal liquid into the rectum. Tell patients to relax and to retain the TCM herbal liquid for at least 2 hours.	Alleviating dysmenorrhea.	Use caution in patients with intestinal lesions.	[97–111]

**Table 2:** Herbal mixture for dysmenorrhea caused by EM treatment in the literature.

Herbal mixture sample/case number (*n*)	Ingredients	Control sample number (*n*)	Total clinical effect rate	Model used	Therapeutic effects and actions	Refs.
Shaofu Zhuyu decoction (SZD); *n* = 20	Xiao Hui Xiang, Gan Jiang, Yuan Hu, Mo Yao, Dang Gui, Chuan Xiong, Guan Gui, Mu Dan Gen, Pu Huang, Wu Ling Zhi	Ibuprofen; *n* = 20	T: 90.00% versus 70.00%	Human study	Alleviating dysmenorrhea. CA-125↓, PEG2↓	[23]
Wengjing decoction (WJD); *n* = 48	Wu Zhu Yu, Mai Dong, Dang Gui, Mu Dan Pi, Chuan Xiong, Ren Sheng, Gui Zhi, E Jiao, Bai Shao, Sheng Jiang, Ban Xia, Gan Cao	Mifepristone; *n* = 48	T: 93.75% versus 79.17%	Human study	Alleviating dysmenorrhea. CD4+↓, CD4+/CD8+↓, IL-4↓, IL-10↓	[26]
Xuefu Zhuyu decoction (XZD); *n* = 60	Dang Gui, Sheng Di Huang, Tao Ren, Hong Hua, Zhi Qiao, Chi Shao, Chai Hu , Gan Cao, Jie Geng, Chuan Xiong, Niu Xi	Mifepristone; *n* = 60	T: 90.0% versus 73.3%	Human study	Alleviating dysmenorrhea. Reducing VAS scores.	[30]
Danggui Sini decoction (DSD); *n* = 37	Dang Gui, Gui Zhi, Shao Yao, Xi Xin, Tong Cao, Zhi Gan Cao, Da Zao	Progesterone; *n* = 37	D: 89.19% versus 67.57%	Human study	Alleviating dysmenorrhea.	[34]
Danggui Shaoyao Powder (DSP); *n* = 40	Dang Gui, Shao Yao, Chuan Xiong, Fu Ling, Ze Xie, Bai Zhu	Progesterone; *n* = 40	T: 97.5% versus 82.5%	Human study	Alleviating dysmenorrhea. Reducing VAS scores. PEG2↓, P↓	[35]

Note: T (Total effect rate) = number of effective cases/total number of cases; where effective case refers to the patients or animal models whose signs and symptoms were improved after treatment. D: dysmenorrhea alleviation rate.

**Table 3:** Chinese traditional patent medicines for treating dysmenorrhea caused by EM.

Chinese traditional patent; sample number (n)	Ingredients	Control; sample number (n)	Total clinical effect rate	Model used	Therapeutic effects and actions	Refs.
Guizhi Fuling capsules (GFC); *n* = 93	Gui Zhi, Fu Ling, Mu Dan Pi, Tao Ren, Bai Shao	Gestrinone; *n* = 94		Human study	CA-125↓, CA-199↓	[52]
ELeng capsules (ELC); *n* = 25	San Leng, E Zhu, Dan Shen, Yu Jin, Bie Jia, Chi Shao, Ji Nei Jin, Zhe Bei Mu	Nemestran; *n* = 25	D: 84.00% -48% vs. 80%-36%	Human study	Alleviate dysmenorrhea CA-125↓, PRL↓	[57]
Dan Bie capsules (DBC)	Dang Shen, San Qi, San Leng, Tao Ren, Dang Gui, Bie Jia, Hai Zao, Du Zhong, Bai Zhu, Ban Zhi Lian, Gui Zhi	GFC		SD rat model	PGF2*α*↓, PGE2↓	[58, 59]
Sanjie analgesic capsules (SAC); *n* = 112	Long Xue Jue, San Qi, Zhe Bei Mu, Yi Yi Ren	Danazol; *n* = 46	T: 92.9% vs. 77.5%	Human study	Alleviate dysmenorrhea Shrink endometrioic lesion Recurrence rate↓	[63]
Dane Fukang paste (DFP); *n* = 75	Dan Shen, E Zhu, Chai Hu, San Qi, Chi Shao, Dang Gui, San Leng, Xiang Fu, Yuan Hu, Gan Cao	Gestrinone; *n* = 75	T: 95.45% vs. 81.82%	Human study	Alleviate dysmenorrhea	[66]

Note: T (Total effect rate) = number of effective cases/total number of cases; effective case refers to the patients or animal models whose signs and symptoms were improved after treatment. D: dysmenorrhea alleviation rate.

**Table 4:** The specifications and efficacy of acupuncture methods.

Therapeutic approach	Specifications	Efficacy	Refs.
Acupuncture	Puncturing a needle into the patient's body at a certain angle.	Relieving pain, reducing serum CA-125, improving clinical symptoms such as irregular menstruation.	[72]
Moxibustion	Burning the dried leaves of mugwort (*Artemisia vulgaris*) to stimulate acupuncture points.	Relieving pain and improving pelvic microcirculation by thermal stimulation.	[76]
Warming acupuncture	Maintaining the position of the needle, twisting the moxa mass around the needle handle to heat it, and transferring the heat into the acupoints through the needle.	Relieving pain, warming meridians, and promoting qi and blood circulation.	[80]
Acupoint catgut embedding	Implanting absorbable catgut into acupoints.	Similar to acupuncture's efficacy, but the stimulation of acupoints is continuous for a few days.	[85]
Electroacupuncture	Addition of electric current to strengthen the stimulating effect of acupuncture on acupoints.	Alleviating inflammatory and neuropathic pain and improving blood circulation.	[87]
Auricular points	Stimulating acupoints distributed on the auricle.	Controlling pain, regulating immunity, and so forth.	[93]

**Table 5:** Acupuncture for dysmenorrhea.

Treatment; sample number (*n*)	Control; sample number (*n*)	Total clinical effect rate	Model used	Therapeutic effects and actions	Refs.
Acupuncture; *n* = 25	Mifepristone; *n* = 25	T: 92.0% vs. 52.0%	Human study	Pain score↓, CA-125↓, recurrence rate↓	[74]
Moxibustion; *n* = 27	Ibuprofen; *n* = 27		Human study	VAS score↓, the days of dysmenorrhea↓	[77]
Acupoint catgut implantation therapy; *n* = 36	Acupuncture; *n* = 36	T: 96.97% vs. 90.63%	Human study	PGF2*α*↓, VAS score↓	[86]
Electroacupuncture; *n* = 36	Mifepristone; *n* = 36	T: 94.4% vs. 91.7%	Human study	Pain score↓, CA-125↓, recurrence rate↓	[90]
Auricular acupuncture; *n* = 37	Herbal decoction; *n* = 30	T: 91.9% vs. 60.0%	Human study	*β*-EP↑, dysmenorrhea score↓	[96]

Note: T (total effect rate) = number of effective cases/total number of cases; effective case refers to the patients or animal models whose signs and symptoms were improved after treatment.

**Table 6:** Main acupoints for the treatment of endometriosis in reports.

The main acupoint	English name	*N*	Percentage (%)
Guanyuan	RN4	117	14.11
Sanyinjiao	SP6	84	10.13
Qihai	RN6	75	9.05
Zhongji	RN3	73	8.81
Zigong	EX-CA1	54	6.51

**Table 7:** CHM enema for dysmenorrhea caused by EM.

Treatment; sample number (*n*)	Control; sample number (*n*)	Total clinical effect rate	Model used	Therapeutic effects and actions	Refs.
Proprietary CHM enema decoction; *n* = 30	Oral Sanjie analgesic capsules; *n* = 30		Human study	CA-125↓, VEGF↓	[100]
CHM enema; *n* = 47	Mifepristone; *n* = 36	T: 89.4% vs. 78.7%	Human study	Pain score↓, recurrence rate↓	[101]
CHM enema, Dihuang Jisheng decoction; *n* = 46	Mifepristone; *n* = 46	T: 91.30% vs. 73.91%	Human study	E2↓, FSH↓, LH↓	[103]
CHM ointments; *n* = 36	Danazol; *n* = 36	T: 94.40% vs. 77.80%	Human study		[107]
CHM enema combined with patch therapy; *n* = 32	Ibuprofen sustained-release capsules; *n* = 32		Human study	VAS score↓	[110]

Note: T (total effect rate) = number of effective cases/total number of cases; effective case refers to the patients or animal models whose signs and symptoms were improved after treatment.

## Data Availability

No data were used to support the findings of this study.
